# The complete chloroplast genome of *Pseudostellaria davidii* (franch.) Pax, 1934

**DOI:** 10.1080/23802359.2023.2195514

**Published:** 2023-04-03

**Authors:** Hongye Zhao, Zhaolei Zhang, Xinyi Li, Yu Tian, Jingyi Zhao, Jinxin Liu, Linchun Shi

**Affiliations:** a Hebei Key Laboratory of Study and Exploitation of Chinese Medicine, Chengde Medical University, Chengde, P.R. China; b Key Laboratory of Chinese Medicine Resources Conservation, State Administration of Traditional Chinese Medicine of the People’s Republic of China, Engineering Research Center of Chinese Medicine Resource of Ministry of Education, Institute of Medicinal Plant Development, Chinese Academy of Medical Sciences & Peking Union Medical College, Beijing, P.R. China

**Keywords:** Pseudostellaria davidii, complete chloroplast genome, Pseudostellaria, phylogenetic analysis

## Abstract

*Pseudostellaria davidii* (Franch.) Pax belongs to subseries distancs of *Pseudostellaria* (Caryophyllaceae), and is mainly distributed in north-eastern Asia. The complete chloroplast (cp) genome of *P. davidii* was assembled and annotated for the first time in this study. The cp genome of *P. davidii* is 149,732 bp in length with the GC content of 36.57%, and it consists of four subregions: a large single-copy (LSC) region of 81,156 bp, a small single-copy (SSC) region of 16,894 bp and two inverted repeats (IR) regions of 25,841 bp each. The cp genome of *P. davidii* encodes a total of 111 unique genes, which are 77 protein-coding genes, four rRNA genes, and 30 tRNA genes. The results of phylogenetic analysis strongly suggested that *Pseudostellaria* was a monophyletic group and *P. davidii* forms an independent sister clade to other species of *Pseudostellaria*.

## Introduction

**Figure 1.**
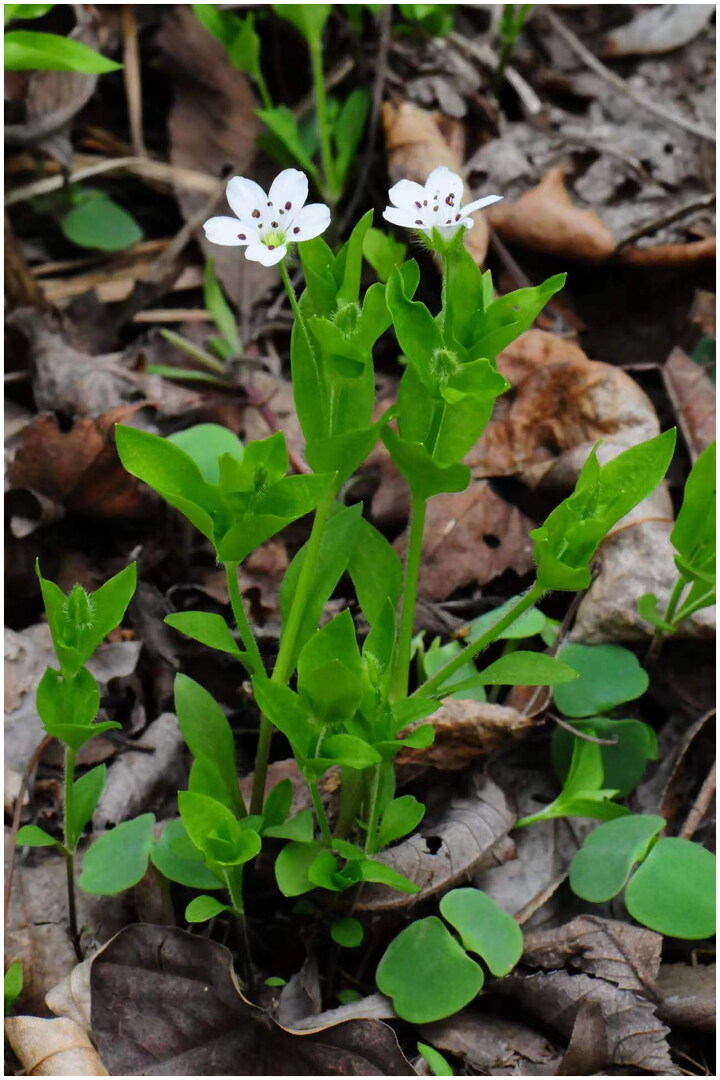
Plant image of *Pseudostellaria davidii.* This photo was taken by Liqiu Zhang with the author’s approval for use.

*Pseudostellaria davidii* was first established by Pax in 1934 as a new species of the tribe Alsineae in Caryophyllaceae and mainly distributed in north-eastern Asia (Pax et al. 1934; Zeng et al. 2016). *P. davidii* was often misidentified due to having minimal external characters to species such as *P. palibiniana* and *P. japonica*. Among *Pseudostellaria*, *P. davidii* has five petals and sepals,10 stamens, two or three styles, and one or two short napiform roots (Figure 1). The root shape and distribution of sepal hairs can be used as diagnostic characters to distinguish *P. davidii* and *P. palibiniana.* The pollen of *P. davidii* has spheroidal grains with 25.76 μm in diameter and 15 round pores with a distance of 6.28 μm apart from each other, which is larger than that of *P. japonica* (Cui et al. 2020). In addition, a natural hybrid between *P. davidii* and *P. palibiniana* has been verified through the analysis of morphological characters, somatic chromosome numbers, pollen sterility, and random amplified polymorphic DNA (RAPD) (Choi et al. 2001). Due to containing valuable information with a highly conservative nature, the complete chloroplast (cp) genome has been widely used in molecular markers, barcode identification, phylogenetic analysis and other fields (Yang et al. 2020; Gu et al. 2022). Therefore, we characterized the structure of the cp genome of *P. davidii* and analyzed its phylogenetic relationship with other species in Caryophyllaceae family.

## Materials and methods

Fresh leaves of *Pseudostellaria davidii* were collected from Tonghua City, Jilin Province (N41°43', E125°56'). The voucher specimen was stored in the herbarium at the Chengde Medical University (http://www.cdmc.edu.cn/, Jinxin Liu, liujx_23@163.com), voucher number is HPAA0142. Total genomic DNA was extracted from *P. davidii* fresh leaves by using the universal genomic DNA extraction kit (Fansheng TCM Technology Co., Ltd, China). The concentration and quality of the extracted DNA were then determined using Qubit 4.0 (Thermo Fisher Scientific, Inc., USA). Genomic DNA was sheared to prepare a PCR-free library of 150 bp. High-throughput sequencing was performed using the Illumina NovaSeq 6000 system, and a total of 2.2 GB pair-end reads was generated. Trimmonmatic v0.38 (Bolger et al. 2014) was employed to remove the adapters and filter low-quality reads. The chloroplast genome was assembled by using the organelle assembler NOVOPlasty v4.2.1 (Dierckxsens et al. 2017). The unique genes of the *P. davidii* chloroplast genome were annotated using CPGAVAS2 web service (Shi et al. 2019). The gene graphical map of the chloroplast genome was constructed using cpgview (http://www.1kmpg.cn/cpgview) (Liu et al. 2022). Then final chloroplast genome of *P. davidii* was submitted to GenBank (Accession number: OP526392).

## Result

**Figure 2.**
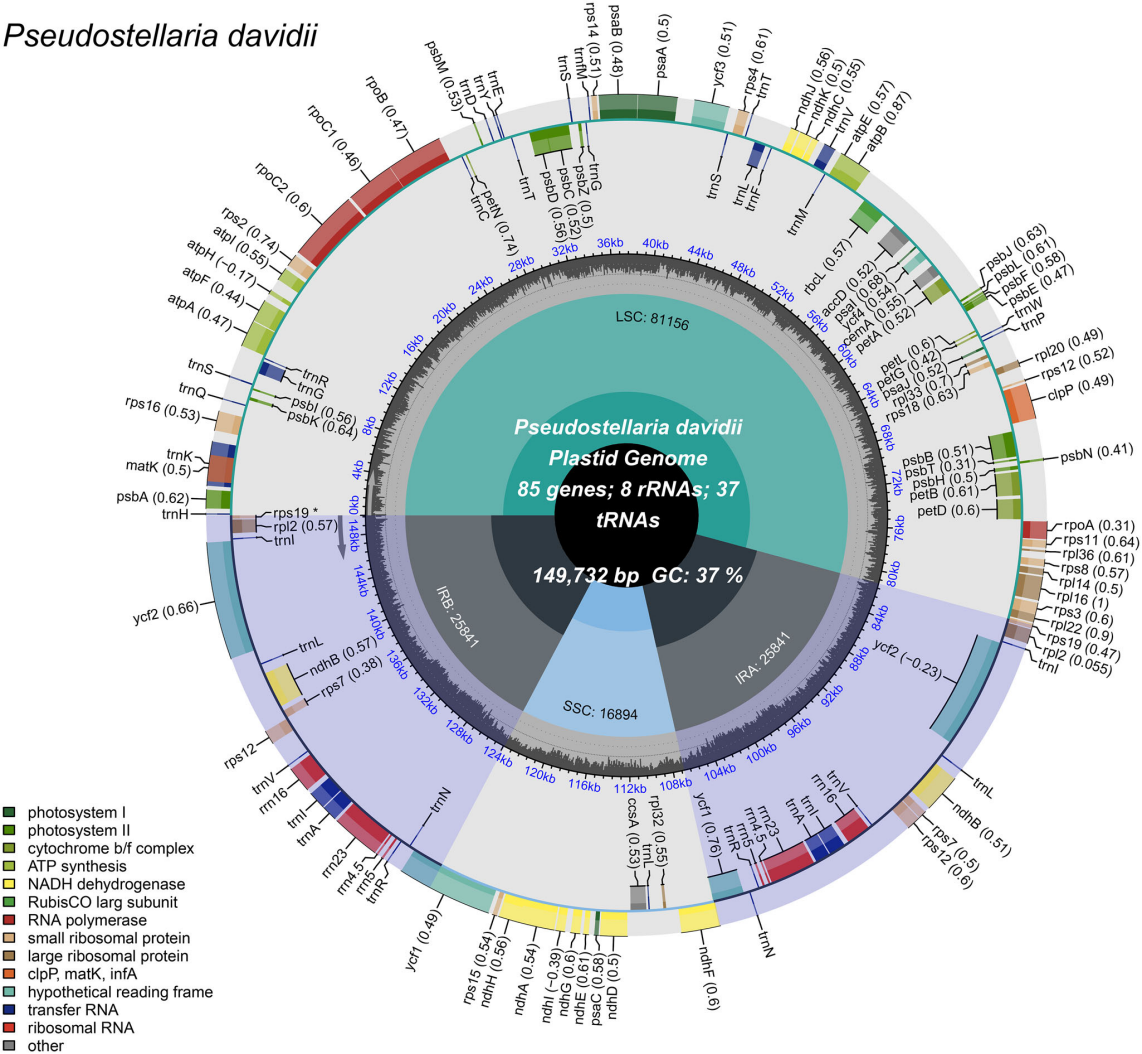
Schematic map of overall features of the *P. davidii* chloroplast genome (Genes drawn outside the outer circle are transcribed clockwise, and those inside are transcribed counter-clockwise. Genes belonging to different functional groups are color-coded. The different colored legends in the bottom left corner indicate genes with different functions. The dark grey inner circle indicates the GC content of the chloroplast genome and the presence of nodes in the LSC, SSC, IR regions).

The chloroplast genome of *Pseudostellaria davidii* is 149,732 bp in length, with an average depth of 794.06X (Supplementary Figure 1). The genome has a conserved quadripartite structure consisting of a large single copy (LSC) region with a length of 81,156 bp, a small single copy (SSC) region of 16,894 bp, and a pair of inverted repeat regions (IRA and IRB) of 25,841 bp each. In total, 111 unique genes were predicted, including 77 protein-coding genes, 30 tRNA genes, and four rRNA genes (*rrn16S*; *rrn23S*; *rrn4.5S*; *rrn5S*) (Figure 2). There were 14 genes (*atpF*, *ndhA*, *ndhB*, *petB*, *petD*, *rpl16*, *rpoC1*, *rps16*, *trnA-UGC*, *trnG-UCC*, *trnI-GAU*, *trnK-UUU*, *trnL-UAA*, and *trnV-UAC*) containing one intron and three genes (*clpP*, *rps12* and *ycf3*) having two introns. Three small-exon genes (*petB*, *petD*, *rps16*) and one trans-spliced gene (*rps12*) were verified to be corrected and annotated with multiple sequence alignment (Supplementary Figure 2).

**Figure 3.**
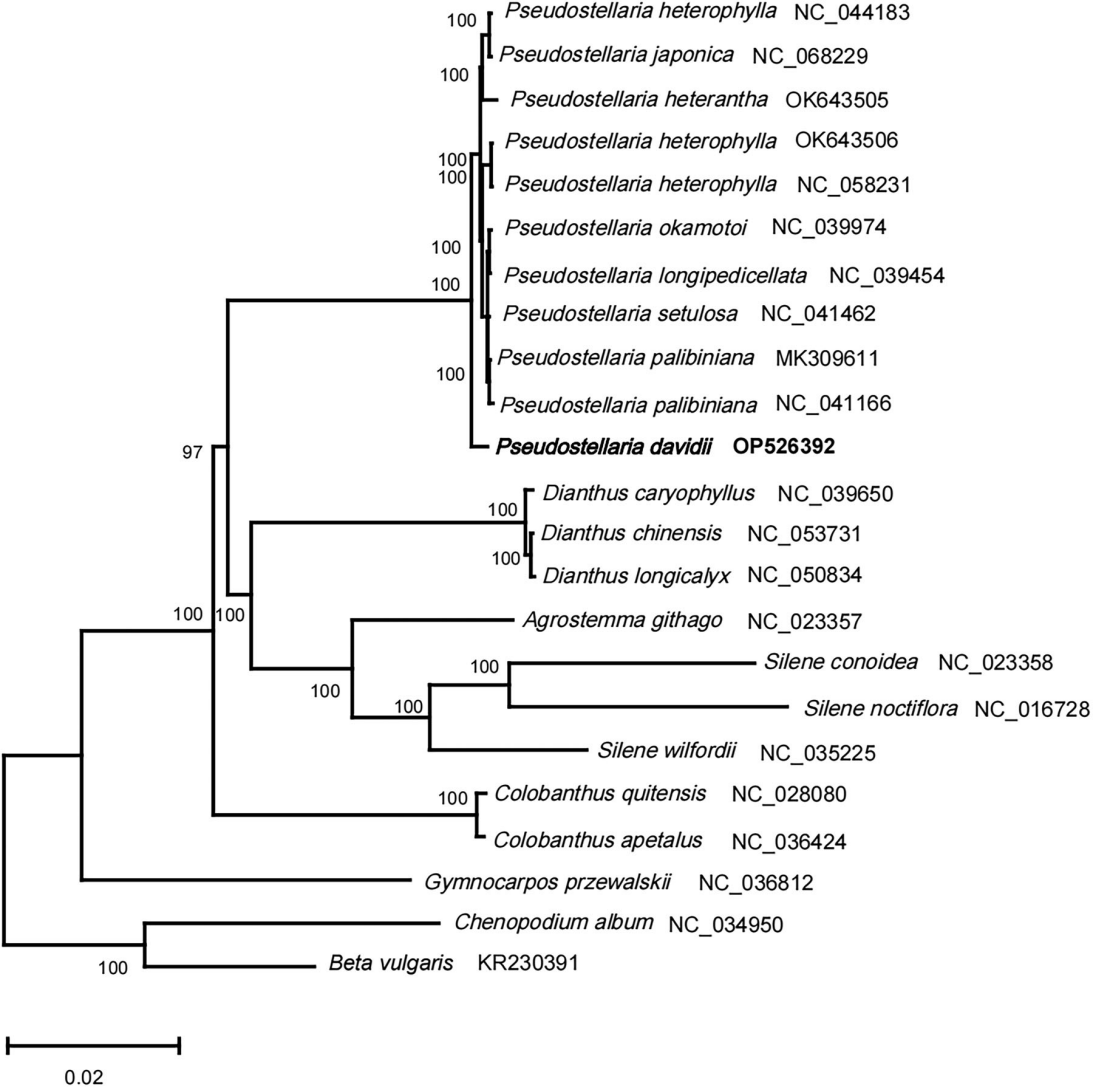
The phylogenetic position for *Pseudostellaria davidii* according to the ML phylogenetic tree constructed based on 23 chloroplast genomes. The following sequences were used: *Pseudostellaria heterophylla* NC_044183 (Kim et al. 2019b), *Pseudostellaria japonica* NC_068229, *Pseudostellaria heterophylla* OK_643505, *Pseudostellaria heterophylla* OK_643506*, Pseudostellaria heterantha* NC_058231*, Pseudostellaria okamotoi* NC_039974, *Pseudostellaria longipedicellata* NC_039454, *Pseudostellaria setulosa* NC_041462, *Pseudostellaria palibiniana* MK_309611 (Kim et al. 2019a), *Pseudostellaria palibiniana* NC_041166, *Dianthus caryophyllus* NC_039650, *Dianthus chinensis* NC_053731, *Dianthus longicalyx* NC_050834, *Agrostemma githago* NC_023357 and *Silene conoidea* NC_023358 (Sloan et al. 2014), *Silene noctiflora* NC_016728 (Sloan et al. 2012), *Silene wilfordii* NC_035225, *Colobanthus quitensis* NC_028080, *Colobanthus apetalus* NC_036424, *Gymnocarpos przewalskii* NC_036812, *Chenopodium album* NC_034950 (Hong et al. 2017), *Beta vulgaris* subsp. *vulgaris* KR_230391 (Stadermann et al. 2015).The sequences used for the tree structure are coding sequences, and the bootstrap support values are shown on the nodes.

Twenty-one complete chloroplast genomes of Caryophyllaceae, and two species from Amaranthaceae (outgroup) were used for constructing Maximum Likelihood (ML) phylogenetic tree (Figure 3) with RAxML v8.2.12 (Stamatakis 2014). The alignment of 73 protein-coding genes was first created using the muscle v5 (Edgar 2022), and then concatenated to a super alignment with a length of 66430 bp. Species of the *Pseudostellaria* genus were clustered together in the phylogenetic trees with a bootstrap of 100, suggesting this genus was a confident monophyletic group. Moreover, *P. davidii* showed an independent sister clade to other species of the genus *Pseudostellaria*. This study provides the cp genome information of *P. davidii*, which would contribute to the species identification and phylogenetic analysis within *Pseudostellaria* and Caryophyllaceae species.

## Discussion and conclusion

In this study, the chloroplast genome sequence of *Pseudostellaria davidii* was assembled for the first time and the structure of this species was annotated. The phylogenetic results indicated that *P. davidii* showed an independent sister clade to other species of genus *Pseudostellaria*, and this study provided new information for the phylogenetic relationship of the Caryophyllaceae family.

## Supplementary Material

Supplemental Material

Supplemental Material

Supplemental Material

## Data Availability

The genome sequence data that support the findings of this study are openly available in GenBank of NCBI at (https://www.ncbi.nlm.nih.gov/) under the accession no. OP526392. The associated BioProject, SRA, and Bio-Sample numbers are PRJNA903544, SRR22352177, and SAMN31807405, respectively.
