# Supplementary figures and images for: The complete chloroplast genome of Pseudostellaria davidii (franch.) Pax, 1934
Source: Mitochondrial DNA B Resour. 2023 Apr 3;8(4):471–4. doi: 10.1080/23802359.2023.2195514 (PMC10071897; doi:10.1080/23802359.2023.2195514)

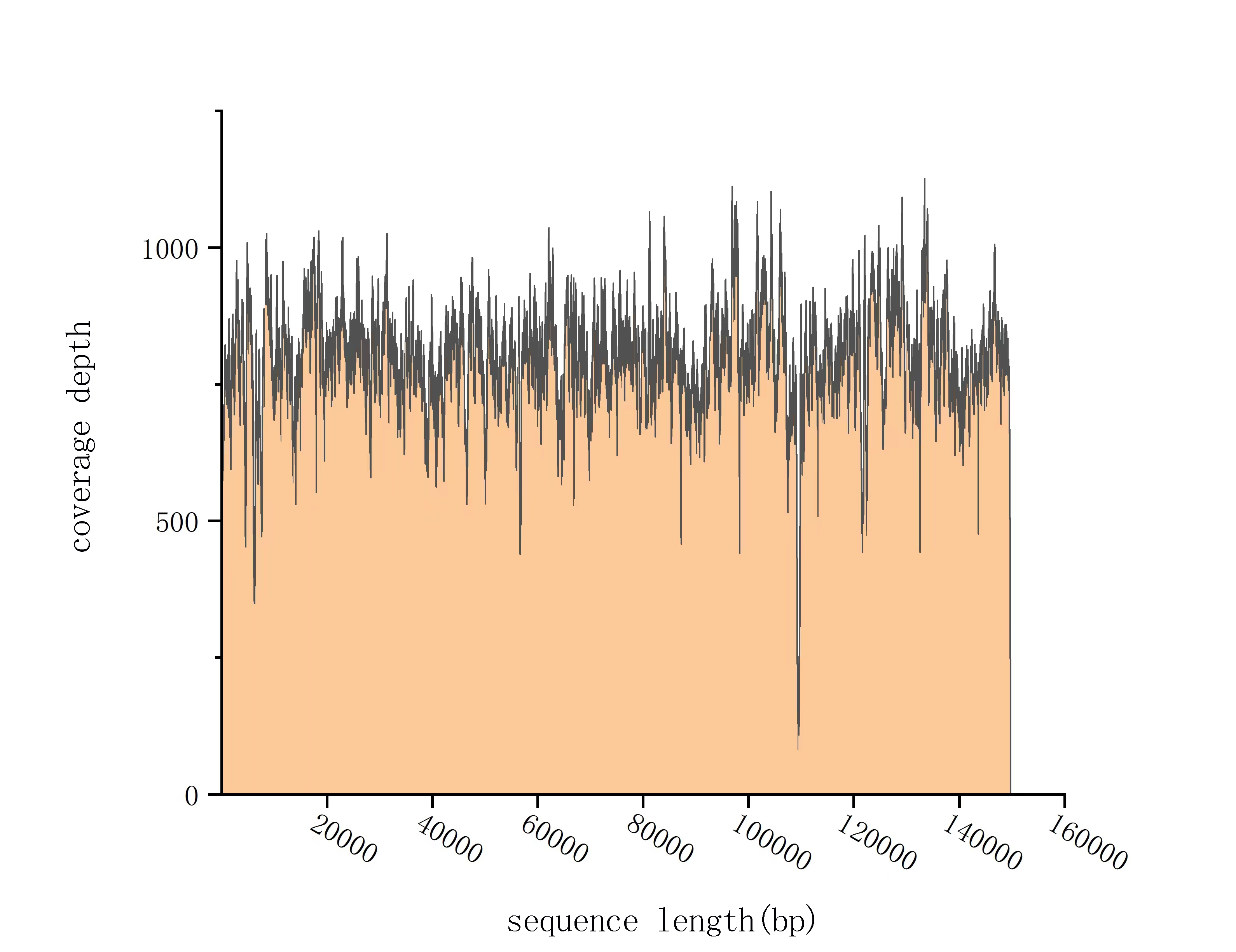

Supplement: Supplemental Material [file TMDN_A_2195514_SM0130.tif]
