# Supplementary material for: The complete chloroplast genome of Pseudostellaria davidii (franch.) Pax, 1934
Source: Mitochondrial DNA B Resour. 2023 Apr 3;8(4):471–4. doi: 10.1080/23802359.2023.2195514 (PMC10071897; doi:10.1080/23802359.2023.2195514)
Supplement: Supplemental Material [file TMDN_A_2195514_SM0070.docx]

**The complete** **chloroplast genome of** ***Pseudostellaria davidii* (Franch.) Pax, 1934**

**Hongye Zhao^a^, Zhaolei Zhang^a,b^, Xinyi Li^a^, Yu Tian^a^, Jingyi Zhao^a^,** **Almaz Boriigidai^c^，Jinxin Liu^a, b*^, Linchun Shi^a,b*^**

a Hebei Key Laboratory of Study and Exploitation of Chinese Medicine, Chengde Medical University, Chengde 067000, China

b Key Laboratory of Chinese Medicine Resources Conservation, State administration of Traditional Chinese Medicine of the People’s Republic of China, Engineering Research Center of Chinese Medicine Resource of Ministry of Education, Institute of Medicinal Plant Development, Chinese Academy of Medical Sciences & Peking Union Medical College, Beijing 100193, China

c Key Laboratory of Ethnomedicine of Ministry of Education, Minzu University of China, Beijing 10081, China

* Correspondence:

Jinxin Liu

liujx_23@163.com

Linchun Shi

linchun_shi@163.com


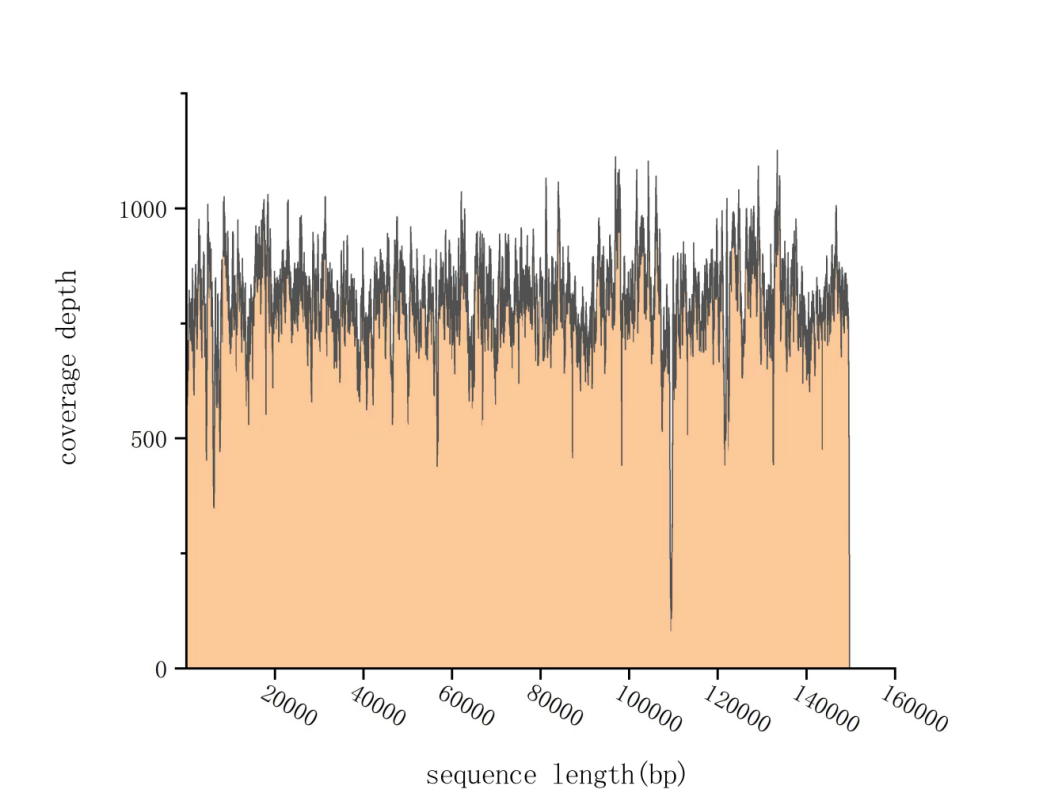


Supplementary Figure 1 Coverage depth figure of the *Pseudostellaria davidii* chloroplast genome. The horizontal coordinate is the base of the chloroplast genome and the vertical coordinate is the depth of sequencing corresponding to that base.


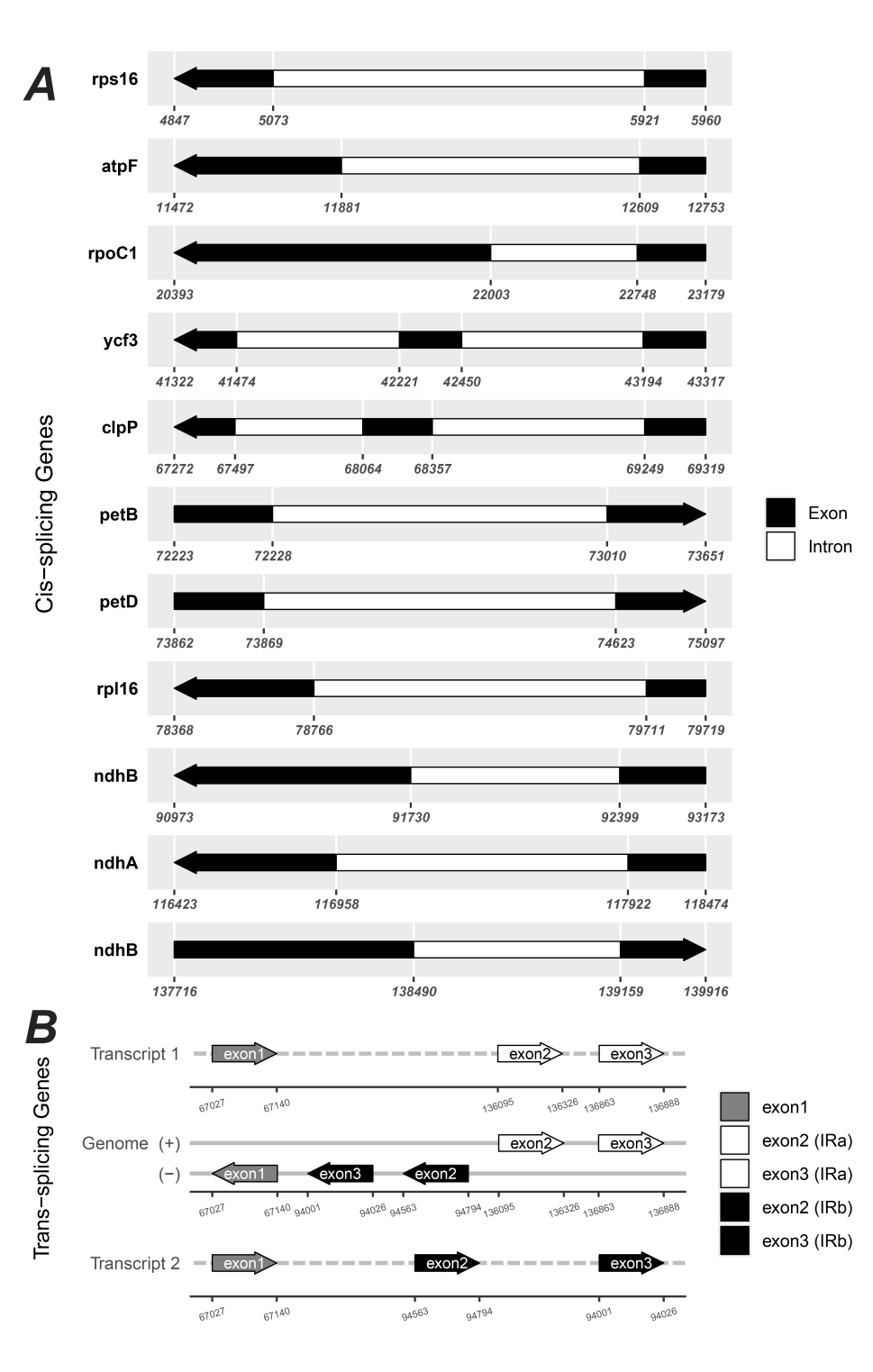


Supplementary Figure 2 Schematic map of the cis-splicing genes in the *Pseudostellaria davidii* chloroplast genome. B. Schematic map of the trans-splicing gene rps12 in the chloroplast genome.
